# Hot Deformation Behavior and Mechanisms of SiC Particle Reinforced Al-Zn-Mg-Cu Alloy Matrix Composites

**DOI:** 10.3390/ma16237430

**Published:** 2023-11-29

**Authors:** Enze Diao, Jianzhong Fan, Zhiyu Yang, Zhaochong Lv, Hao Gao, Junhui Nie

**Affiliations:** 1 GRINM Group Corporation Limited, National Engineering & Technology Research Center for Non-Ferrous Metals Composites, Beijing 101407, China; diaoenze9334@163.com (E.D.); jzfan@grinm.com (J.F.); 2 GRINM Metal Composites Technology Co., Ltd., Beijing 101407, China; yangzhiyu@grinm.com (Z.Y.); lvzhaochong@grinm.com (Z.L.); gaohao@grinm.com (H.G.); 3 General Research Institute for Nonferrous Metals, Beijing 100088, China

**Keywords:** aluminum matrix composite, constitutive equation, hot-processing maps, microstructure evolution, fracture failure mechanism

## Abstract

A systematic and comprehensive analysis of the hot deformation and mechanisms of SiC particle-reinforced aluminum matrix composites is significant for optimizing the processing of the composites and obtaining the desired components. Based on this, related research on 11 vol% SiCp particle-reinforced 7050Al matrix composites was carried out. Hot compression experiments were carried out on the Gleeble-3500 thermal simulator to study the hot deformation behavior of composites at the temperature of 370–520 °C and strain rate of 0.001–10 s^−1^. The hyperbolic sine constitutive equation of the material was established, and the processing map was calculated. Combining the typical metallograph and misorientation angle distribution, the microstructure evolution mechanism of composites was analyzed, and the effect of particles on recrystallization behavior was investigated. Under certain process conditions, the dominant deformation mechanism of composites changed from dynamic recovery (DRV) to dynamic recrystallization (DRX), and the grain boundary sliding mechanism began to play a role. In addition, high temperature tensile and elongation at break were tested, and it was found that the dominant form of fracture failure changed from brittle fracture of the particles to ductile fracture of the matrix as the temperature increased.

## 1. Introduction

Particle-reinforced metal matrix composites (PRMMCs) are commonly used in aerospace and new energy vehicles due to their high modulus, strength, light density, and fatigue resistance compared to traditional alloys [1,2,3,4]. Various ceramic reinforcements have been added to the development of composites, including Al_2_O_3_, SiC, TiB_2_ and Al_3_Ti [5,6,7,8,9]. Among them, SiC particles have attracted widespread attention in industries because of their high elastic modulus, low cost, and excellent resistance to corrosion [10,11]. Currently, SiC particle-reinforced aluminum matrix composites from Al-Cu-Mg-Zn system by powder metallurgy (PM) is an effective strategy for the industrial production of PRAMCs, which have high strength, strong interface bonding, and sufficient economic feasibility [12,13]. However, the presence of SiC particles prevents uniform load distribution within composites, limiting their plastic deformation capacity [14,15,16,17]. Obtaining the structural components without defects and non-homogenized microstructure is still difficult.

To enhance the mechanical properties of composites, hot-working processing techniques, such as hot rolling [18], hot extrusion [19], and die forging, [20] can be employed. In recent years, extensive research has been conducted on the hot deformation behavior of composites, which has provided fundamental theoretical guidance for their hot working [21,22,23,24]. Generally, particles can stimulate nucleation on recrystallization, making the composites form a uniform and fine recrystallization microstructure during deformation [3,25]. The addition of TiC-TiB_2_ particles in an Al-Zn-Mg-Cu-based composite, according to a study conducted by Liu et al. [26], increases the proportion of low-angle grain boundaries (LAGBs) and high-angle grain boundaries (HAGBs). This promotes dislocation multiplication, which helps maintain the high strength and plasticity of the extruded profiles of composites. Wang et al. [27] conducted a study on the failure mechanism of an in situ composite made of a 7075 aluminum matrix reinforced with TiB_2_ particles. They found out that at a low temperature and high strain rate, the composite experiences particle fracture and interface debonding. Apart from the stress state, the internal failure of components is also influenced by softening processes like recovery and recrystallization [28]. However, the previous research on the hot deformation behavior of composites are studied mostly through theories more suitable for single-phase homogeneous materials, without fully investigating the effect of reinforcement particles on the microstructure evolution of composites [29,30]. In addition, considering that the deforming components are manipulated by complicated states of stress, temperature and strain rate, a comprehensive study about the effect of deformation processing conditions on the deformation behavior and failure mechanism of composites is necessary.

In this paper, isothermal compression tests at various temperatures, from 370 to 520 °C, and strain rates, from 0.001 to 10 s^−1^, and high-temperature tensile tests were conducted on SiCp/Al-Zn-Mg-Cu composites. A constitutive equation with the Arrhenius equation and processing maps were established, and the microstructure and micro-fractography were examined. This work aims to provide a comprehensive understanding of the influence of different processing processes on the microstructure evolution, mechanical properties, deformation behaviors, and failure mechanism of composites. Therefore, it can support the development and processing of new particle-reinforced metal matrix composites.

## 2. Materials and Methods

**Figure 1:**
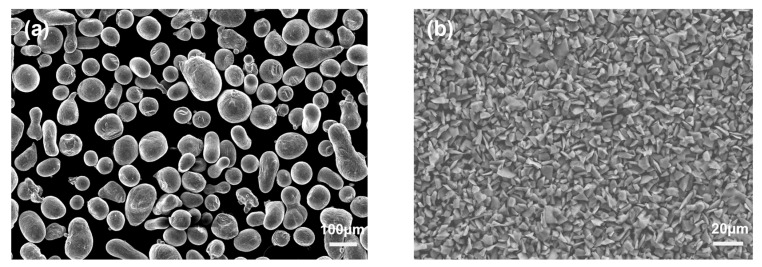
SEM images of (**a**) raw alloy powders and (**b**) raw α-SiC powders.

The 11 vol% SiCp/7050Al composites were prepared by the powder metallurgy approach. α-SiC particles with 99.9% purity and an average diameter of 12 μm were used as reinforcements, as shown in Figure 1a. Composites were designed based on the Al-Zn-Mg-Cu alloy, and the chemical compositions of the alloy are listed in Table 1. Pure gas-atomized spherical alloy powders with 99.9% purity and an average diameter of 120 μm were used, as shown in Figure 1b.

In this study, cylindrical composite specimens with the height of 15 mm and the diameter of 10 mm were prepared, and in order to reduce the deformation friction and ensure the accuracy of the data, the specimens were ground until Ra = 1.6, and graphite flakes were padded on both ends of the specimens. The specimens were compressed isothermally using the Gleeble-3500 simulator (Dynamic Systems Inc., Poestenkill, NY, USA) up to a total true strain of 0.6 with temperatures of 370, 400, 430, 460, 490, and 520 °C and strain rates of 0.001, 0.01, 0.1, 1, and 10 s^−1^. The specimens were heated at a rate of 5 °C/s, held for 3 min to unify the temperature distribution and then quenched to facilitate observation of the microstructure after deformation. The SiC particles in the specimen before deformation were uniformly distributed in the matrix, and after deformation, the particles were still determined to be macroscopically uniformly distributed in the matrix without affecting the testing of mechanical properties.

The tension specimens with a gauge diameter of 5 mm and a length of 30 mm were machined, and uniaxial tensile tests were operated using AMSLER-100-20 universal testing machine (ZwickRoell, Shanghai, China) at a constant strain rate of 3 × 10^−3^ s^−1^, at temperatures of 20, 100, 150, 200, 300, 350 °C. The elongation was measured using a clip-on extensometer.

The deformed samples were cut along the direction of centerline and were subjected to mechanical grinding and Cross Section Polisher (CP). Typical metallographs of the samples were obtained by optical microscopy (OM). The microstructure of the composites was photographed using a JSM-7800F Scanning Electron Microscope (SEM) (Japan Electron Optics Laboratory Co., Ltd., Tokyo, Japan) equipped with an X-ray Energy Dispersive Spectroscopy (EDS) module (Oxford Instruments Technology (Shanghai) Co., Shanghai, China), and information on grains and grain boundaries was obtained.

## 3. Results

### 3.1. Flow Stress Behavior

**Figure 2:**
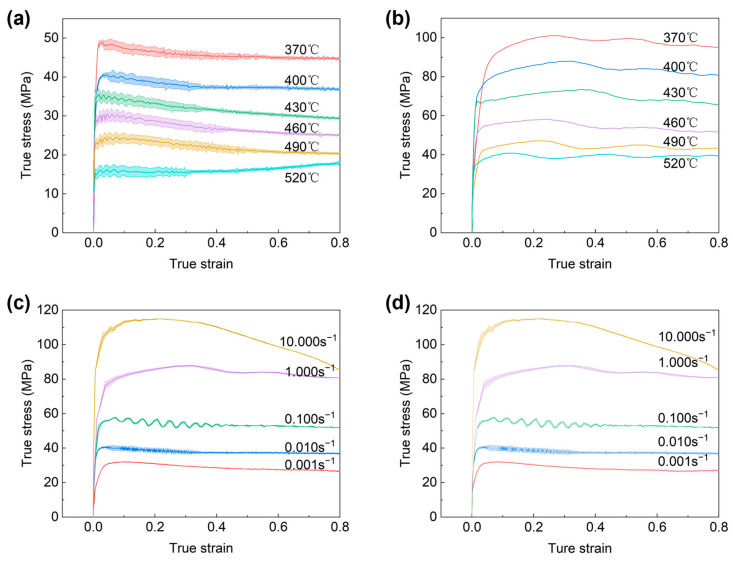
True stresstrue strain curves of the composites at different temperatures and strain rates. (**a**) Strain rate equal to 0.01 s^−1^, different temperatures; (**b**) Strain rate equal to 1 s^−1^, different temperatures; (**c**) Temperature equal to 400 °C, strain rate different; (**d**) Temperature equal to 40 °C, strain rate different.

The true stress–true strain curves of the 11% SiC/7050Al composites at different temperatures and strain rates are shown in Figure 2. The curves clearly reflect the effects of deformation conditions on the peak stresses and final steady-state stresses. The flow stresses appear to change significantly with strain rate and temperature, indicating that the hot deformation behavior of the composites is sensitive to the deformation conditions.

In fact, the deformation of composites is regulated by a combination of work hardening and dynamic softening mechanisms. At the initial stage of strain increase, the true stress increases sharply, forming a peak due to the restriction of reinforcement particles on the dislocation slip. And then the stresses reach a state of approximate equilibrium and decrease slightly due to the reduction in dislocation density and the restructuring of dislocations, resulting from dynamic recovery (DRV) or dynamic recrystallization (DRX).

In addition, the flow curves fluctuate dramatically in the initial stage, which is a typical repeated recrystallization phenomenon. It is because of the presence of rigid SiC particles, which makes a large number of dislocation stacks at the interface between SiC particles and the alloy during hot deformation, and providing sufficient distortion energy and ideal nucleation locations for the occurrence of dynamic recrystallization. As the temperature rises, the diffusion ability of atoms is enhanced, dislocations can climb and slide easily, grain boundary sliding occurs more frequently, and dynamic softening is enhanced. In contrast, as the strain rate increases, the composites will not have sufficient time for dynamic softening.

However, it is not easy to define the deformation mechanism of the composites by the analysis of flow curves only. The evolution of microstructure needs to be further investigated by constructing the processing maps and constitutive equations.

### 3.2. Constitutive Equations

Constitutive equations describe the relationship between flow stress σ, temperature T, and strain rate $\dot{\varepsilon }$. Sellars and Tegar proposed an Arrhenius equation that considers the deformation activation energy Q and temperature T. The equation plays a pivotal role in comprehending the conduct of materials when exposed to diverse conditions, and its importance is particularly pronounced in the fields of metallurgy and material science.

(1) $$\dot{\varepsilon }=f(\sigma )\exp\left(-\frac{Q}{RT}\right)$$

Arrhenius equation in hyperbolic sinusoidal form can be used at any stress level:

(2) $$\dot{\varepsilon }=A{\left[\sinh\left(\alpha \sigma \right)\right]}^{n}\exp\left(-\frac{Q}{RT}\right)$$

When the stress is at a low level, i.e., ασ < 0.8:

(3) $$\dot{\varepsilon }=A_{1}\sigma^{n_{1}}\exp\left(-\frac{Q}{RT}\right)$$

When the stress is at a high level, i.e., ασ > 1.2:

(4) $$\dot{\varepsilon }=A_{2}exp(\beta \sigma )\exp\left(-\frac{Q}{RT}\right)$$

where A, A_1_, A_2_, α, β, n, and n_1_ are constants and α = β/n_1_; Q is the deformation activation energy; R is the gas constant; T is the temperature; and σ is the flow stress.

The influence of the strain rate and temperature on flow stress during deformation can also be quantified using the Zener–Hollomon parameter. This parameter holds the physical significance of a temperature-compensated deformation rate factor, and it is expressed as the Z-H parameter.

(5) $$Z=\dot{\varepsilon }\exp\left(\frac{Q}{RT}\right)$$

**Figure 3:**
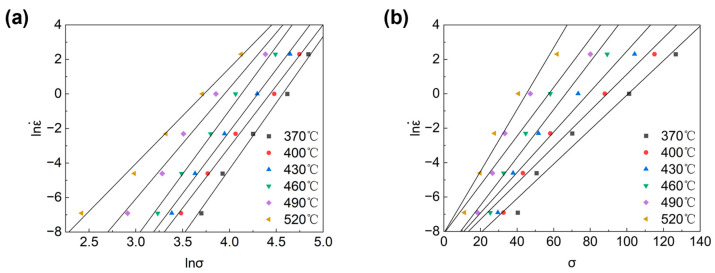
The relation between strain rate and flow stress: (**a**) ln$\dot{\varepsilon }$ versus lnσ, (**b**) ln$\dot{\varepsilon }$ versus σ.

The slopes of the curves ln$\dot{\varepsilon }$-lnσ and ln$\dot{\varepsilon }$-σ can be determined by taking the logarithms of both sides of Equations (3) and (4) when the temperature is certain. These slopes correspond to n_1_ and β, respectively, which in turn can be used to find α at different temperatures. Figure 3 illustrates the relationship between ln$\dot{\varepsilon }$lnσ and ln$\dot{\varepsilon }$-σ. The slopes of each set of slopes can be found and averaged to obtain n_1_ = 6.882, β = 0.13057, and α = 0.01897.

Taking the natural logarithmic differentiation of both sides of Equation (2) yields:

(6) $$Q=R{\left\{\frac{\partial ln\dot{\varepsilon }}{\partial \ln\left[\sinh\left(\alpha \sigma \right)\right]}\right\}}_{T}{\left\{\frac{\partial ln[sinh(\alpha \sigma )]}{\partial (1/T)}\right\}}_{\dot{\varepsilon }}$$

**Figure 4:**
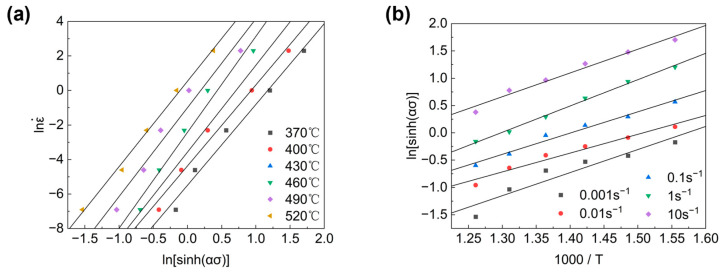
Relationship between (**a**) flow stress and strain rate; (**b**) flow stress and deformation temperature.

According to Equation (6), the deformation activation energy Q equals the product of the gas constant R, slope of the $ln\dot{\varepsilon }$-$\ln\left[\sinh\left(\alpha \sigma \right)\right]$ slash and slope of the $\ln\left[\sinh\left(\alpha \sigma \right)\right]$-(1/T) slash. The relationship between flow stress and strain rate, and the relationship between flow stress and deformation temperature are shown in Figure 4.

Substituting Equation (2) into Equation (5) gives the following:

(7) $$Z=A{\left[\sinh\left(\alpha \sigma \right)\right]}^{n}$$

Taking the logarithms on both sides of Equation (7) gives the following:

(8) lnZ=lnA+nln[sinh⁡(ασ)]

**Figure 5:**
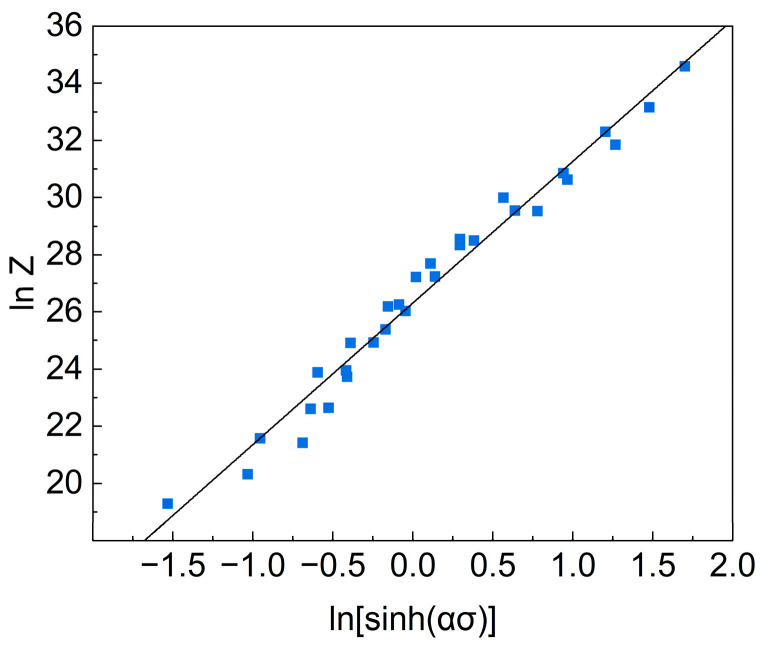
Relationship between parameter Z and stress.

The corresponding strain rates and corresponding peak stresses at different temperatures are brought into Equation (8), and the corresponding lnZ-ln [sinh(ασ)] relation curves are plotted as shown in Figure 5.

Substituting the required parameters into Equation (2), the stress–strain constitutive equation for the experiment is obtained.

(9) $$\dot{\varepsilon }=2.66*10^{11}{\left[\sinh\left(0.018973\sigma \right)\right]}^{5.0101}\exp\left(-\frac{172.744}{RT}\right)$$

The constitutive equation can also be described in terms of the Z parameter:

(10) $$\sigma =52.715ln\left\{{\left(\frac{Z}{2.66*10^{11}}\right)}^{\frac{1}{5.0101}}+{\left[{\left(\frac{Z}{2.66*10^{11}}\right)}^{\frac{1}{5.0101}}+1\right]}^{\frac{1}{2}}\right\}$$

### 3.3. Processing Maps

The dynamic materials model (DMM) processing map is a commonly used tool to explain the deformation mechanism of composites and predict their processability under different deformation conditions. According to the DMM model, the total power dissipation during hot deformation is divided into two parts: G represents the power dissipation due to hot deformation, and J represents the power dissipation due to microstructure evolution.

(11) $$P=\int_{0}^{\dot{\varepsilon }}\sigma d\dot{\varepsilon }+\int_{0}^{\sigma }\dot{\varepsilon }d\sigma =G+J$$

where P represents the total power dissipation, ε represents the strain rate, and σ represents the stress. J can be represented by the following expression:

(12) $$J=\int_{0}^{\sigma }\dot{\varepsilon }d\sigma =\frac{m}{m+1}\sigma \dot{\varepsilon }$$

From Equation (11), the strain rate sensitivity parameter (m) can be revealed as follows:

(13) $$m={\left(\frac{\partial J}{\partial G}\right)}_{T,\varepsilon }=\frac{\dot{\varepsilon }d\sigma }{\sigma d\dot{\varepsilon }}={\left[\frac{\partial (\ln\sigma )}{\partial (\ln\dot{\varepsilon })}\right]}_{T,\varepsilon }$$

The nondimensional power dissipation efficiency (η) is a measure that represents the proportion of power dissipated solely by the microstructure evolution process during hot deformation, in comparison to the total power dissipated.

(14) $$\eta =\frac{J}{J_{max}}=\frac{2m}{m+1}$$

In addition, the temperature sensitivity parameter (s) was also introduced to assist in the analysis of the research, and m is defined as follows:

(15) $$s=\frac{1}{T}\frac{\partial \ln\sigma }{\partial (1/T)}$$

**Figure 6:**
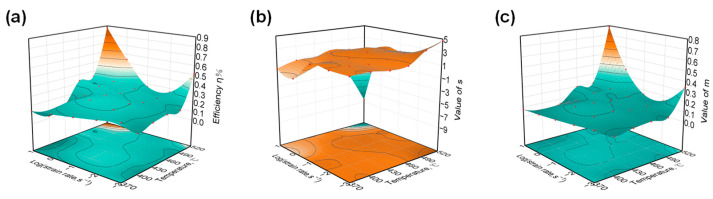
Three-dimensional curves of (**a**) strain rate–efficiency η%–temperature; (**b**) strain rate–value of s–temperature; (**c**) strain rate–value of m–temperature.

During hot deformation, changes in the microstructure and deformation mechanism of the composites cause a macroscopic flow stress response, which is reflected by changes in the value of s, m, and η. The values of η, s, and m for 11% SiCp/7050Al composites at different temperatures and strain rates are shown in Figure 6 below.

**Figure 7:**
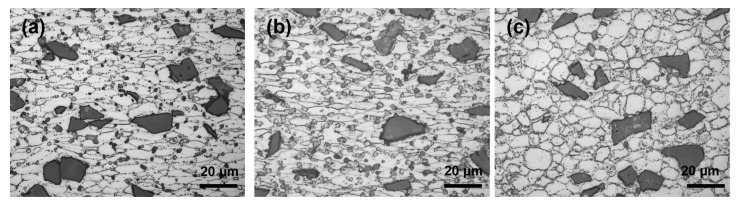
Microstructures of the deformed specimens compressed under different conditions: (**a**) 370 °C, 0.001 s^−1^; (**b**) 370 °C, 0.1 s^−1^; (**c**) 490 °C, 0.001 s^−1^. Dark areas are SiC particles, light areas are aluminium alloy matrix.

In the interval of strain rate of 0.001–0.1 s^−1^ and temperature of 370–460 °C, the values of power dissipation (η) and strain rate sensitivity parameter (m) are low, and the value of temperature sensitivity parameter (s) is basically unchanged. Based on the above characteristics, it can be inferred that the microstructure evolution mechanism in this region is dynamic recovery (DRV). From the microstructure analysis, in the hot-pressing specimen with strain rate of 0.001 s^−1^ and temperature of 370 °C in Figure 7a, the original grains in the aluminum alloy matrix are elongated into fibers, and the grain boundaries are parallel to the direction of the shear force, which is the DRV mechanism (Table 2).

At a strain rate of 1 s^−1^, there is a noticeable inflection point in the temperature sensitivity parameter (s). This is because the deformation mechanism based on diffusion does not have enough time to take effect at high strain rates. As a result, the flow stresses show low sensitivity towards temperature changes.

And as shown in Figure 7b, many rounded and smooth fine grains with obvious sliding deformation characteristics appeared at the grain boundary, indicating that the sliding deformation mechanism of the grain boundary began to play a role at this time, and that the microstructure evolution mechanism at this time changed to the dynamic recrystallization mechanism (DRX). 

Only at higher strain states did the matrix alloy undergo dynamic recrystallization, which is attributed to the presence of the Al prior particle boundary (PPB) at the grain boundaries of the matrix alloy, and the existence of SiC-reinforced phases. These two factors work together to pin the grain boundaries and prevent grain boundary migration strongly.

When the temperature reaches around 480 °C, both the η and s values show inflection points, and from the microstructure analysis, in the hot-pressing specimen with a strain rate of 0.001 s^−1^ and temperature of 480 °C in Figure 7c, the microstructure has been completely occupied by isometric new grains. It can be inferred that 480 °C is the end recrystallization temperature.

The change in the value of the power dissipation factor is not enough to adequately determine the unsafe processing zone during thermal processing; it also needs to be combined with the basis of rheological instability to determine the region of rheological instability. The basis of instability ξ is defined as follows:

(16) $$\xi \left(\dot{\varepsilon }\right)=\frac{1}{T}\;\frac{\partial \ln\left(\frac{m}{m+1}\right)}{\partial \ln\dot{\varepsilon }}+m<0$$

**Figure 8:**
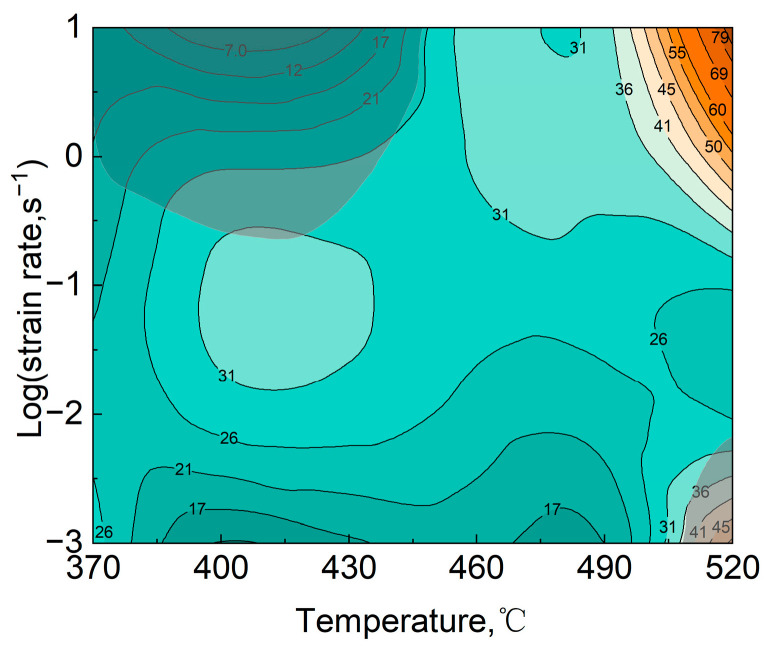
Hot processing map of the composites at the true strain of 0.6.

The hot-processing map can be obtained by superimposing the destabilization map and the power dissipation map. The hot-processing map of 11% SiCp/7050Al at 60 strain constructed on the basis of the above theory is shown in Figure 8 below. Two destabilization regions appear, which are the high-velocity low-temperature region (370–450 °C, 0.3–10 s^−1^) and the low-velocity high-temperature region (510–520 °C, 0.001–0.01 s^−1^).

In Figure 8, the temperature of 370–480 °C, the strain rate of 0.001–0.1 s^−1^ region, the power dissipation value is more moderate, and there is no risk of instability, the composite material in this region has a good plastic deformation ability and steady-state rheological stress, has a better processing performance, the region should be the best thermal processing region for composite materials.

## 4. Discussion

### 4.1. Effects of Processing Processes on Microstructure Evolution

Processing process conditions play a decisive role in the microstructure evolution of composites, but microstructure observation by metallograph alone is unable to accurately characterize some low-angle grain boundaries (LAGBs) and substructures, and thus the judgment of recovery and recrystallization of composites is not accurate enough. In this study, the microstructure evolution mechanism of SiCp/Al composites during deformation is precisely analyzed by equip.

**Figure 9:**
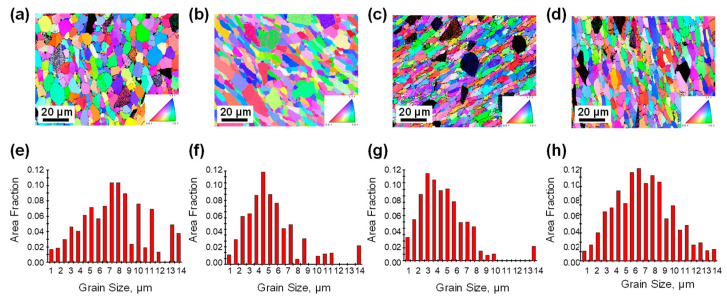
Grain structures of the SiCp/7050Al samples. (**a**–**d**) Reconstructed inverse pole figure maps of the SiCp/7050Al samples: (**a**) no compression, (**b**) 430 °C, 0.001 s^−1^, (**c**) 430 °C, 1 s^−1^, (**d**) 520 °C, 1 s^−1^, respectively; (**e**–**h**) corresponding grain size distributions of (**a**–**d**).

Figure 9a shows the EBSD observations of the specimens before hot compression, and Figure 9b,d shows the EBSD observations of the specimens after hot compression at 430 °C, 0.001 s^−1^ and 520 °C, 1 s^−1^, respectively. And it can be seen that the grains of the latter two are elongated, and the average grain sizes (2.14 μm, 2.10 μm) are increased with respect to the original conditions, where the average grain sizes is 1.59 μm, but no large number of recrystallized grains were found. This is because at 430 °C, 0.001 s^−1^, the deformation rate is slow, the grains have sufficient time to deform, the slip motion of dislocations within the grains can be sufficiently carried out, the density of dislocations within the grains is low, and the dislocations are not enough to rearrange to form subcrystalline boundaries. At 520 °C, 1 s^−1^, due to the existence of softening effect of high temperature on grain boundaries, relative sliding of some grain boundaries can be induced to coordinated plastic deformation. Figure 9c shows the EBSD observation results of the specimens after hot-pressing at 430 °C, 1 s^−1^, and it can be seen that a large number of equiaxed recrystallized grains appeared in the matrix alloy, and the average grain size (1.39 μm) was reduced compared with the average grain size (1.59 μm) under the original condition. This is due to the formation and migration of HAGBs driven by stored energy to form new grain structures in the deformed Al matrix, and recrystallization behavior occurs.

The misorientation angle distribution is an important piece of information reflecting the internal structure of the material, and the comparative analysis of the misorientation angle distribution in the matrix alloy under different thermal processing parameters can also be used to infer the microstructure evolution mechanism during the plastic deformation of the composite material.

**Figure 10:**
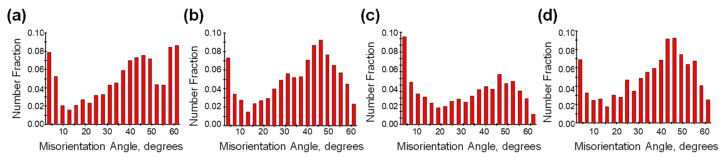
Misorientation angle distribution of composites in different processing processes: (**a**) no compression, (**b**) 430 °C, 0.001 s^−1^, (**c**) 430 °C, 1 s^−1^, (**d**) 520 °C, 1 s^−1^.

Figure 10a–d shows the misorientation angle distribution of composites before hot compression; after hot-pressing at 400 °C, 0.001 s^−1^ parameter; after hot-pressing at 400 °C, 1 s^−1^ parameter; and after hot-pressing at 520 °C, 1 s^−1^ parameter, respectively. As shown in Figure 10c, the proportion of small-angle grain boundaries (LAGBs, <5°) increases significantly after hot-pressing at 400 °C, 1 s^−1^ parameter, which is due to a large number of dislocations generated during plastic deformation under this processing condition, and dislocations are rearranged to form the boundary of subgrain, and the boundary of subgrain absorbs newly generated dislocations and gradually transforms them to the slightly higher angle of the LAGBs but still does not exceed the boundaries of the low-angle grain boundaries; the matrix of the proportion of high-angle grain boundaries (HAGBs, >15°) in the alloy decreases relative to that before hot-pressing, again due to the formation of a large number of new LAGBs, resulting in a decrease in the proportion of HAGBs.

The statistics of the misorientation angle distribution are shown in Figure 10. The average misorientation angle of the grain boundaries after hot compression at 400 °C, 1 s^−1^ parameter is 30.6°, which is 6.274° lower than that before hot compression, and the proportion of small-angle grain boundaries is 13.3%, which is 5.4% higher than that before hot compression, which confirms that the base alloys underwent a large degree of geometrodynamic recrystallization behavior under this parameter.

From the above analysis, it can be seen that both temperature and strain rate play a key role in influencing the microstructure evolution mechanism of the matrix during the thermal processing of composites. The grain boundaries in the grains tend to be more difficult to produce sliding under the double pinning effect of the silicon carbide particles and the pristine aluminum powder boundaries. The relatively low temperature and relatively fast strain rate can make the dislocation density in the matrix alloy greatly improved, and the dislocations continue to be multilateralized and transformed into equiaxed subcrystals and fine equiaxed grains inside the deformed grains.

### 4.2. Effects of Processing Processes on Failure Mechanism

**Figure 11:**
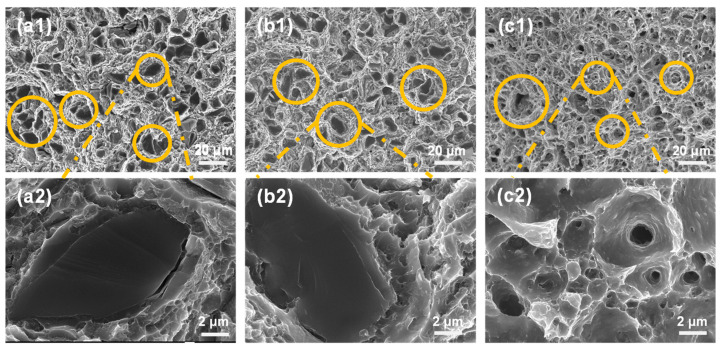
SEM photographs of tensile fracture of composites at different temperatures: (**a1**) room temperature, 500×. (**b1**) 150°C, 500×. (**c1**) 300°C, 500×. (**a2**) room temperature, 5000×. (**b2**) 150 °C, 5000×. (**c2**) 300 °C, 5000×. The areas with typical fracture characteristics are circled by the yellow circle, and the dashed lines guides an enlarged view of this localised area.

The SEM photographs of the tensile fracture of the composites at room temperature, 150 °C, and 300 °C are given in Figure 11. For room temperature tensile fractures, there are a large number of tough nests in the fracture of the composite, and the composite is characterized by ductile fracture. During the tensile process, most of the SiC particles fractured and created a smooth surface at the fracture point. This indicates that the external force was effectively transmitted to the SiC particles through the Al matrix. Hence, it can be inferred that the interfacial bonding strength between the Al matrix and the SiC particles was higher, and that the strength of the aluminum matrix was also high during this time. 

When the temperature is increased to 150 °C high-temperature stretching, the situation is similar to the room temperature stretching, and the toughness fracture characteristics of the aluminum matrix are still dominated, with a large number of tough nests distributed on the tearing prongs, and basically no particles are dislodged. However, the degree of fragmentation of SiC particles at this time is weaker than that at room temperature stretching, indicating that the interfacial bonding strength is still high at this time, but the strength of the Al matrix decreases with the increase in temperature.

In the case of high-temperature tensile fracture at 350 °C, the presence of crushed particles was hardly found at the fracture, and there were a large number of tough nests and cavities in the matrix, most of which existed independently of each other, and there was no problem regarding the merging of the cavities leading to the failure of the material. In the case of high-temperature stretching, the strength of the matrix alloy decreases to a certain extent due to the presence of deformation mechanisms, such as dynamic recovery and dynamic recrystallization, at which time matrix-induced damage becomes the dominant factor in the failure of the composite material.

**Figure 12:**
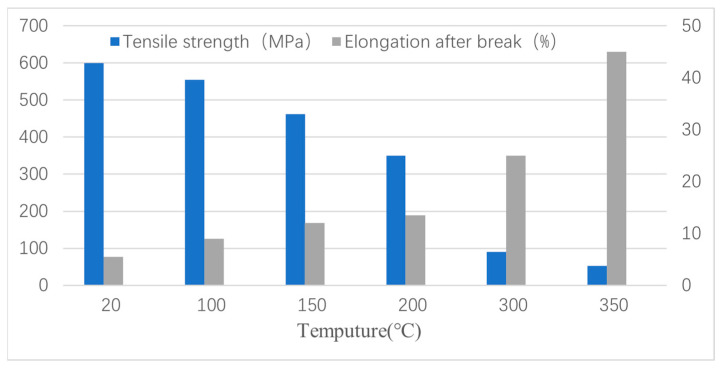
Tensile strength and elongation after break of composites at different temperatures: room temperature, 100 °C, 150 °C, 200 °C, 300 °C, and 350 °C.

As the temperature increases, the degree of fracture of the SiC-reinforced phase particles decreases and the number of fractured particles also decreases, while the number of extracted particles increases. This is due to the fact that the strength of the matrix alloy gradually decreases with increasing temperature, and the bonding of SiC-reinforced particles with the interface of the alloy matrix becomes weaker and weaker. As can be seen from Figure 12, with the increase in temperature, the tensile strength of the composites gradually decreases and the elongation at break gradually increases, indicating that the fracture failure form of composites is dominated by the brittle fracture of the particles, and gradually transformed to the toughness fracture of the matrix alloy as the dominant fracture.

It can be seen that the effect of temperature on the tensile properties and fracture mechanism of composites is significant.

## 5. Conclusions

In this paper, 11 vol.% SiC-particle-reinforced Al-Zn-Mg-Cu alloy matrix composites were prepared by the powder hot-pressing method, and the hot deformation behaviors of the composites at 370–520 °C, 0.001–10 s^−1^, were investigated. The influences of the processing processes on the microstructure evolution and failure forms of the composites were systematically analyzed, and the following conclusions were drawn:

(1) The thermal compressive rheological stress σ of composites satisfies the hyperbolic sinusoidal rheological stress constitutive equation with the deformation temperature T and strain rate ε:

$$\dot{\varepsilon }=2.66*10^{11}{\left[\sinh\left(0.018973\sigma \right)\right]}^{5.0101}\exp\left(-\frac{172.744}{RT}\right)$$

(2) In the processing diagram of true strain 0.6, two destabilization regions appear, which are the high-velocity low-temperature region (370–450 °C, 0.3–10 s^−1^) and the low-velocity high-temperature region (510–520 °C, 0.001–0.01 s^−1^). At the strain rate of 1 s^−1^, the microstructure evolution mechanism changed from dynamic recovery (DRV) to the dynamic recrystallization mechanism (DRX).

(3) Recrystallization occurs during hot-pressing of the composites, and 480 °C is the end recrystallization temperature. Right when dynamic recrystallization occurred at 400 °C, 1 s^−1^, the average misorientation angle distribution within the composites decreased by 6.274° compared to that before hot compression, and the proportion of small-angle grain boundaries increased by 5.4% compared to that before hot compression.

(4) The fracture failure form of composites is significantly affected by temperature. As the temperature increases, the dominant form of fracture failure in composites changes from brittle fracture of the particles to the ductile fracture of the matrix alloy.

## Figures and Tables

**Table 1:** Chemical compositions of alloy powder (wt.%).

Element	Zn	Mg	Cu	Zr	Si	Fe	Others	Al
	6.2	2.2	2.4	0.12	0.10	0.09	0.13	Bar.

**Table 2:** Misorientation angle information statistics for composites.

Misorientation Angle	>15°	<10°	<5°	Average
No compression	81.1%	15.2%	7.9%	36.855
400 °C, 0.001 s^−1^	82.5%	13.5%	7.3%	35.460
400 °C, 1 s^−1^	68.2%	24.4%	13.3%	30.581
520 °C, 1 s^−1^	82.9%	12.7%	6.9%	35.741

## Data Availability

Data are contained within the article.
